# The Lawsonia Paradox: The Hidden Burden of Silent Disease

**DOI:** 10.3390/pathogens15070743

**Published:** 2026-07-15

**Authors:** Umberto Rolla, Fabio Persico, Giovanbattista Guadagnini, Antonio Caleffi, Annalisa Scollo

**Affiliations:** 1 Martini S.p.A, 47020 Longiano, FC, Italy; u.rolla@martinigruppo.com; 2 Independent Researcher, 20900 Monza, MB, Italy; fpersic@tin.it; 3 Vet Evolution, 26010 Campagnola Cremasca, CR, Italy; gioguadagnini@gmail.com; 4 Independent Researcher, 46026 Quistello, MN, Italy; antoniocaleffi@gmail.com; 5 Department of Veterinary Sciences, University of Turin, 10095 Grugliasco, TO, Italy

**Keywords:** *Lawsonia intracellularis*, pig, bacterial disease, prevention, vaccination, management

## Abstract

*Lawsonia intracellularis*, the etiologic agent of porcine proliferative enteropathy (PPE), represents a growing challenge for modern swine production systems. The infection often occurs in a subclinical form and, together with limited awareness of its true production impact, may delay diagnosis and the timely adoption of control measures. In our opinion, this under-recognition of subclinical infection represents the greatest barrier to successful PPE control. Disease management is further complicated by frequent co-infections, limitations of currently available diagnostic tools, and the progressive reduction in routine antimicrobial use, which has revealed the true burden of PPE under commercial conditions. We therefore argue that vaccination should no longer be considered a stand-alone preventive measure, but rather the cornerstone of an integrated, farm-specific management strategy combining accurate diagnostic interpretation, optimized production flows, biosecurity, nutritional management, and continuous veterinary–farmer collaboration. Drawing on both published evidence and the long-term field experience of the authors, this Opinion discusses the key challenges limiting effective PPE control and proposes a practical framework to improve disease recognition, prevention, and sustainable management under commercial production conditions.

## 1. Introduction

*Lawsonia intracellularis* is an obligate anaerobic intracellular bacterium that primarily infects the small intestine and, more rarely, the large intestine of pigs and other animal species, including hamsters and horses. The infection is characterized by epithelial proliferation, hemorrhage, necrosis, or a combination of these lesions, and is commonly referred to as porcine proliferative enteropathy (PPE). The disease has a significant impact on both animal health and productive efficiency in swine herds [1].

Transmission occurs mainly via the fecal–oral route [1]. After reaching the ileal crypts, *L. intracellularis* selectively infects immature enterocytes, within which it penetrates and replicates intracellularly. The bacterium disrupts intestinal epithelial homeostasis by modulating the β-catenin/Wnt and Notch signaling pathways, thereby inhibiting normal enterocyte differentiation, reducing goblet cell maturation, and maintaining crypt cells in a proliferative state. These molecular alterations ultimately result in the characteristic epithelial hyperplasia that defines porcine proliferative enteropathy [1,2,3].

Histological alterations include crypt hyperplasia, mucosal thickening, and reduced epithelial maturation [2], which underline the clinical manifestations of the disease, including acute hemorrhagic forms in severe cases [4]. Although PPE most commonly affects growing pigs [2], *L. intracellularis* is considered endemic in swine populations [3]. This widespread distribution is also supported by European epidemiological studies; for example, a herd-level prevalence of 65.7% and a within-herd prevalence of 51.5% were reported in Poland [5].

Defining the true extent of infection remains challenging, as the disease may occur in a subclinical form [2]. In this regard, an observational field study conducted by Stege et al. [4] documented the presence of the pathogen in herds without detectable clinical signs, highlighting that *L. intracellularis* can circulate even in the absence of overt disease. Diagnostic complexity is further increased by concurrent factors that may modify disease expression, such as co-infection with other enteric pathogens [6].

In addition, piglets are particularly vulnerable during the post-weaning period, when the gastrointestinal tract undergoes profound structural and functional changes associated with the transition from milk to solid feed and the maturation of intestinal defenses. This transitional phase represents a critical window during which the intestine is more susceptible to the development of enteric disorders [7,8]. Overall, infection with *L. intracellularis* continues to represent a major challenge for the swine industry, being associated with substantial productive and economic losses [2,9].

Taken together, these observations explain why, in this Opinion, the term “silent disease” extends beyond the subclinical form of PPE. It encompasses the overall tendency of *L. intracellularis* infection to remain under-recognized under commercial conditions, whether because infection is subclinical, presents as mild or chronic disease with non-specific clinical signs, is masked by concurrent enteric conditions, or results primarily in hidden productive and economic losses rather than overt clinical outbreaks.

Considering the clinical and productive impact of PPE and the complexity of its clinical and diagnostic interpretation, vaccination remains one of the main disease control strategies. Currently, two commercial vaccines against *L. intracellularis* are available: an oral live attenuated vaccine (Enterisol^®^ Ileitis, Boehringer Ingelheim International GmbH, Ingelheim-Rhein, DE, USA) and an injectable inactivated vaccine (Porcilis^®^ Lawsonia, Merck Animal Health, USA Inc., Rahway, NJ, USA).

This paper focuses on the oral live vaccine and on field outcomes associated with its use, because it has generated the largest body of longitudinal field evidence currently available, especially in endemic production systems. Its longer availability on the Italian market has also provided extensive practical experience, allowing long-term assessment of its impact under field conditions. In this context, oral vaccination has demonstrated beneficial benefits at the clinical and productive levels, as supported by field studies conducted both in Italy and abroad. For example, Almond and Bilkei [10] reported significant improvements in productive performance in vaccinated pigs. More recent data from similar studies in Italy further confirmed these findings, showing an improvement in feed conversion ratio (from 3.32 to 3.23; *p* = 0.017), a reduction in mortality (from 3.93% to 2.82%; *p* < 0.001), and a marked decrease in antimicrobial treatments for enteric disorders (−80.14%) in vaccinated groups [11]. The effectiveness of oral vaccination was further supported by a meta-analysis of field studies published in the proceedings of an international scientific conference in 2022, which estimated a net economic benefit of USD 8.30 per vaccinated pig after accounting for vaccine costs [12].

Beyond these documented advantages, vaccination has also demonstrated positive effects in complex clinical settings characterized by enteric co-infections. Nevertheless, despite the progress achieved, relevant challenges in disease management remain mainly related to the underestimation of the impact of subclinical infections and the limitations of current diagnostic approaches [4,6]. Vaccination alone cannot fully compensate for inadequate management conditions or delayed disease recognition. Effective control of PPE requires integration of vaccination strategies with timely diagnostics, appropriate production flow management, biosecurity measures, and nutritional support tailored to the specific epidemiological conditions of each herd.

In our opinion, current discussions on *L. intracellularis* still focus predominantly on treatment and overt clinical disease, whereas the greatest health and economic burden arises from subclinical infections that remain undetected until production losses become evident. We therefore argue that vaccination should not be viewed as an isolated preventive intervention, but as a cornerstone of an integrated herd-health strategy combining early diagnostics, biosecurity, optimized production flow, and nutritional management. This perspective emerged from a structured exchange among Italian veterinarians with extensive experience in swine health management, aimed at identifying the main barriers to effective PPE control under commercial production conditions and developing a shared interpretation of the available evidence. Throughout this Opinion, published scientific evidence is integrated with the authors’ collective long-term field experience. Whenever robust evidence is available, our conclusions are supported by the literature; where evidence remains limited, the recommendations presented reflect the authors’ interpretation based on their collective field experience, providing a practical framework for improving disease recognition, prevention, and control.

## 2. Discussion

Porcine proliferative enteropathy can manifest in three main clinical forms: acute, chronic, and subclinical. Among these, the subclinical form is by far the most prevalent in modern swine production systems [3] and is associated with a measurable negative impact on productive performance, as also demonstrated under experimental conditions [13]. Beyond its direct effects on productivity, subclinically infected pigs may act as a reservoir for *L. intracellularis*, contributing to pathogen dissemination within the group and favoring the persistence of infection at the herd level. This silent circulation represents one of the major challenges in the effective control of PPE, as performance losses may occur in the absence of overt clinical signs.

Based on these premises, the following sections discuss the key diagnostic, management, and economic aspects associated with PPE, with a specific focus on the implications of subclinical infection for disease recognition, decision-making, and control strategies in endemic production systems.

### 2.1. Diagnostic Challenges and Underestimation of the Clinical Impact of Lawsonia intracellularis

In our experience, one of the main challenges associated with both chronic and subclinical forms of porcine proliferative enteropathy is the presence of mild, nonspecific, or even absent clinical signs, which can lead to a substantial underestimation of the true impact of the disease. This frequently results in a reduced perception of health risk, even in the presence of relevant enteric alterations, making disease recognition difficult without targeted and systematic monitoring.

In addition, the presence of co-infections can significantly modify disease expression. In commercial production systems, *L. intracellularis* frequently coexists with pathogens such as *Brachyspira* spp., *Salmonella* spp., and porcine circovirus type 2 (PCV2), all of which may contribute to enteric disease and impair productive performance. As a result, clinical manifestations—including diarrhea, reduced growth, and increased within-group variability—are often non-specific, making it difficult to identify the primary etiologic agent based on clinical signs alone. This aspect, well recognized in field practice, is also supported by the scientific literature, which highlights how the overlap of clinical manifestations among different enteropathies can delay the achievement of an accurate diagnosis [1,6].

### 2.2. Challenges in Uniquely Linking Diagnostic Findings, Clinical Expression, and Economic Damage

In swine herds, the concurrent circulation of multiple enteric pathogens can substantially modify the evolution and severity of intestinal lesions, often resulting in clinical and pathological patterns that exceed those attributable to *L. intracellularis* alone. Based on our field experience, co-infection with pathogens such as *Brachyspira* spp. should be regarded as a major modifier of disease expression, often amplifying enteric damage and complicating diagnostic interpretation. This view is supported by a controlled experimental study suggesting an additive effect in co-infected animals [14]. A potential interaction has also been proposed for *Salmonella*, as in vitro studies have highlighted a possible synergistic mechanism between *L. intracellularis* and *Salmonella enterica* serovar *Typhimurium* [15]. Consistent with these observations, clinical experience indicates that effective control of *L. intracellularis*, achieved through vaccination, contributes to improved enteric stability at the group level, a condition often associated with reduced circulation pressure of other enteric pathogens. This interpretation is supported by evidence from both controlled experimental and observational field studies. An experimental coinfection study demonstrated reduced fecal shedding of *Salmonella* [16] in pigs orally vaccinated against *L. intracellularis*, while an observational field study reported a lower serological prevalence of *S. enterica* [17]. These findings are particularly relevant given that *Salmonella enterica* remains one of the major health challenges for the swine industry, with a significant prevalence also reported in finishing pigs destined for slaughter [18].

In our opinion, interpreting the impact of *L. intracellularis* under commercial conditions requires a broader herd-level perspective. In real-life production settings, the overall damage observed cannot be attributed solely to the presence of concomitant pathogens. Additional factors, including exposure to toxic substances, the presence of inflammatory mediators, and suboptimal management conditions, may further exacerbate intestinal damage and clinical expression, thereby increasing the complexity of diagnostic interpretation and decision-making.

### 2.3. Impact of Lawsonia intracellularis Infection on Replacement Breeding Animals

In our experience, replacement gilts represent a particularly vulnerable category with respect to *L. intracellularis* infection, especially in the period following introduction into the herd, when adequate immunity has not yet been established [4]. During this phase, infection may manifest in acute and sometimes severe forms, particularly when animals are exposed to highly virulent strains [19]. We believe that this susceptibility is often underestimated in commercial production systems, despite its potentially important consequences for both animal health and future reproductive performance. In the absence of adequate control measures or vaccination, the risk of developing acute disease increases substantially.

One of the most characteristic clinical signs in this context is the presence of fresh or partially digested blood in the feces, which may appear suddenly even in apparently healthy gilts and result in significant economic losses, especially when disease onset occurs prior to first insemination [4]. This vulnerability is particularly evident in herds that regularly purchase replacement gilts, where achieving homogeneous immunization at group level is more challenging, with potential consequences for both animal health and productive performance [10].

In animals destined for reproduction, even subclinical forms of infection may negatively affect growth trajectories. Reduced or irregular growth can delay the achievement of target body weight and, consequently, the onset of puberty, ultimately compromising future reproductive performance [20]. Based on our experience in Italian commercial heavy pig production, this effect is often more readily observed in animals not selected for entry into the breeding herd according to national production schemes—such as those applied in Italian Protected Designation of Origin (PDO) pork systems—where only a subset of gilts meeting specific productive and conformational criteria is retained as future breeders. These non-selected animals are therefore redirected to conventional grow–finish batches for slaughter production. This unique management practice provides an opportunity to recognize the subtle impact of subclinical *L. intracellularis* infection, which may become apparent as a relative growth disadvantage—slower and more variable weight gain compared with pen-mates managed under the same conditions—despite the absence of overt clinical signs [12,20].

Based on our field experience, the progressive reduction in routine antimicrobial use has not only changed the management of PPE but has also revealed the true impact of subclinical enteric diseases that had previously been partially masked by metaphylactic treatments. From a management perspective, particularly in smaller production systems, PPE has traditionally been addressed through therapeutic interventions aimed at limiting immediate economic losses [2]. However, this approach is increasingly difficult to reconcile with modern production models, which are progressively oriented toward preventive strategies and a reduction in antimicrobial use. In this context, and in line with the current European regulatory framework, the gradual decrease in routine antibiotic administration has made the circulation of enteric pathogens such as *L. intracellularis*, as well as other enteric pathogens including *Brachyspira* spp., more evident [9].

Field observations support this dynamic, as acute forms of ileitis tend to emerge more frequently in herds characterized by minimal or absent antimicrobial use and, paradoxically, in high-health-status production systems [4,20]. For these reasons, there is a growing need to adopt alternative management strategies based on preventive measures, including strengthened biosecurity and the implementation of selective, targeted therapeutic interventions.

### 2.4. Economic Impact of Lawsonia intracellularis Infection and Key Barriers to Effective Disease Control

The economic impact of *L. intracellularis* infection arises from both direct and indirect losses, each of which is highly relevant at herd level. Direct losses, which are more readily quantifiable, include costs associated with therapeutic treatments and mortality-related losses [12,20]. Indirect losses, although more difficult to measure, are often equally significant and primarily involve reduced feed efficiency, impaired productive performance, lower weight gain, and, most importantly, decreased batch uniformity [20]. Based on our field experience, batch uniformity is one of the most underestimated economic consequences of subclinical *L. intracellularis* infection and represents a key economic parameter, as it directly affects carcass classification and the homogeneity of carcass weights—factors that are increasingly valued along the pork production chain [20]. In this context, even limited variability in growth performance may translate into substantial economic penalties.

With specific reference to chronic and subclinical forms of the disease, there remains a lack of robust and up-to-date data reflecting current production systems. Available estimates often fail to account for critical variables such as genetic background and antimicrobial management strategies [4,9]. Nevertheless, existing literature provides useful reference points to contextualize the economic burden of subclinical infection. An early estimate presented in conference proceedings by McOrist suggested a minimum loss of approximately EUR 3 per grow–finish pig, a figure that, although not specific to the Italian context, may reasonably serve as a benchmark for comparable production scenarios [21].

A further challenge we consistently encounter in field practice relates to the difficulty of systematically collecting reliable and standardized production data. This limitation substantially affects the objective evaluation of vaccination effectiveness across different production systems, particularly in light of the marked heterogeneity among farms. We therefore believe that effective PPE control cannot be based solely on conventional productive indicators, but it requires a broader approach that integrates management practices, antimicrobial use patterns, and health status.

From this perspective, disease control strategies must also consider the ethical and practical implications of antimicrobial use [9], the potential advantages associated with participation in production schemes promoting responsible antimicrobial stewardship—such as the Italian Eco-schema 1, a national incentive framework linked to antimicrobial reduction and animal welfare standards [22]—and the increasing market-driven risks of exclusion from antibiotic-free supply chains in the event of inadequate health management [23]. Adopting a comprehensive and integrated perspective that incorporates these dimensions is therefore essential to develop sustainable PPE control strategies aligned with current regulatory frameworks and evolving market expectations.

### 2.5. Limited Awareness of the Strategic Importance of Prevention and Control

As field veterinarians, we have consistently perceived that one of the main obstacles to the effective control of *L. intracellularis* infection is the persistent underestimation of the strategic importance of prevention, observed both among farmers and, in some cases, among veterinarians. This insufficient level of awareness may reduce interest in continuous professional development and limit the adoption of available control strategies [24,25]. As a consequence, infection is often identified only at a late stage, typically following targeted indications provided by the herd veterinarian. This issue is further compounded by the fact that most of the farms do not have the structural and managerial conditions required for reliable measurement of productive parameters affected by the disease, thereby complicating both detection and monitoring of infection at herd level [26].

Within this context, prevention clearly represents the most effective strategy for the management of *L. intracellularis* infection. Among the available preventive measures, vaccination remains a particularly relevant tool, contributing to improved overall herd efficiency and to a reduction in costs associated with disease management [12,16].

In Italy, vaccine coverage is currently estimated at approximately 20%, according to aggregated national market data (personal communication); however, a progressive increase in vaccine use has been observed over the last five years. This trend appears to be driven by increased awareness of the disease, improved diagnostic capabilities, and growing attention toward management practices aimed at limiting antimicrobial use, in line with current regulatory and stewardship-oriented frameworks [9,27].

## 3. Priority Actions for the Control of Porcine Proliferative Enteropathy

To ensure effective control of *L. intracellularis* infection, theoretical awareness based solely on international data is not sufficient to foster active engagement among stakeholders. In our opinion, communication strategies must be supported by concrete and context-specific data, tailored to the national production landscape and derived from scientific publications, technical reports, and field observations. Within this framework, targeted training plays a pivotal role, as it strengthens the competencies required for effective disease management. Investing in continuous professional development programs therefore becomes a priority, enabling veterinarians to remain consistently updated and to translate evolving knowledge into practical decision-making. Another key aspect concerns the decision-making dynamics between farmers and veterinarians, which are often grounded in long-standing relationships of trust. This relationship represents a privileged channel for the exchange of objective data and for facilitating the adoption of innovative control strategies. In this context, the field veterinarian plays a strategic role. Through direct knowledge of the farm reality, the veterinarian is uniquely positioned to tailor interventions to the specific needs of each production system and to accurately monitor their outcomes, thereby ensuring highly qualified clinical and managerial support.

### 3.1. Adequate Training on Diagnostic Protocols

In our opinion, optimal management of PPE requires a tailored diagnostic approach, adapted to the specific health status and management conditions of each farm. In this context, there is a clear need for tools capable of accurately and early identifying the presence of *L. intracellularis*, supported by a thorough understanding of the available diagnostic methods and the correct interpretation of their results.

From an applied perspective, both clinical and laboratory investigations play a central role. Diagnostic tools such as serology and histological examination, complemented by biomolecular analyses, represent valid options, although they require the support of specialized laboratories. Correct interpretation of results—particularly in cases of seropositivity—is essential to avoid misinterpretation and to appropriately guide therapeutic or preventive decisions. This need becomes especially evident in cases of persistent enteric disorders, where subclinical infections may easily go unnoticed. In such situations, we consider fecal sampling and PCR analysis as a useful first-line tool, which should be integrated with further investigations to achieve a comprehensive diagnostic assessment.

According to the authors, future studies should further explore the role of quantitative PCR, which could complement and refine current diagnostic protocols, as already suggested by existing evidence in the literature [25]. Accurate diagnosis is essential because *L. intracellularis* frequently coexists with other enteric pathogens that may produce similar clinical manifestations. Since the available vaccine is pathogen-specific and does not confer protection against other enteric pathogens, an imprecise diagnosis may lead to inappropriate control measures and limit the overall effectiveness of herd health interventions.

### 3.2. Tailored Vaccination: A Key Element for Effective Control

To ensure maximum effectiveness, vaccine positioning must be carefully defined according to the specific productive conditions of each farm. This requires a thorough analysis aimed at accurately determining both the timing and the modality of administration, identifying when infection is most likely to occur and adapting the vaccination protocol accordingly. In everyday practice, however, the decision to implement a vaccination plan is often influenced by the degree of urgency attributed to the intervention by the herd veterinarian. This assessment depends on several factors, including the overall health and productive status of the herd and the priority assigned to PPE in relation to other concurrent conditions. For this reason, vaccination should not be considered a stand-alone intervention, but rather part of a broader herd health strategy integrating diagnostics, biosecurity, nutritional management, and optimized production flows.

### 3.3. Appropriate Nutritional Approach

Field experience confirms that appropriate nutritional management plays a decisive role in maintaining intestinal functionality. Diet composition directly influences the structure and activity of the gut microbiome, contributing to the establishment of a more stable gastrointestinal microenvironment. This relationship is also supported by the scientific literature, which indicates that different dietary regimens can markedly modulate intestinal microbial communities [7,8]. In the specific case of *L. intracellularis*, dietary factors have been shown to influence the severity of infection and the expression of intestinal lesions, further highlighting the role of nutrition as a modulating factor in disease outcome [28].

### 3.4. Biosecurity and Production Flow Management

Biosecurity represents a fundamental component in the management of *L. intracellularis* infection, as measures implemented in this area are aimed at preventing both the introduction and the within-farm spread of the pathogen, thereby contributing to a reduction in both disease incidence and clinical severity. An effective biosecurity plan includes thorough sanitation of facilities, controlled management of animal movements, and limitation of contact with potential external sources of infection. These measures should be complemented by proper organization of production flows. In particular, the application of all-in/all-out systems and careful planning of sanitary downtime periods are essential to interrupt pathogen transmission between different animal groups. These practices are especially relevant in herds characterized by high hygienic and sanitary standards, where limited previous exposure to pathogens may reduce herd immunity and favor the emergence of acute clinical forms. Consistent with this scenario, the scientific literature suggests that an integrated approach combining biosecurity measures and vaccination represents the most effective strategy for the control of porcine proliferative enteropathy [19,20].

**Figure 1:**
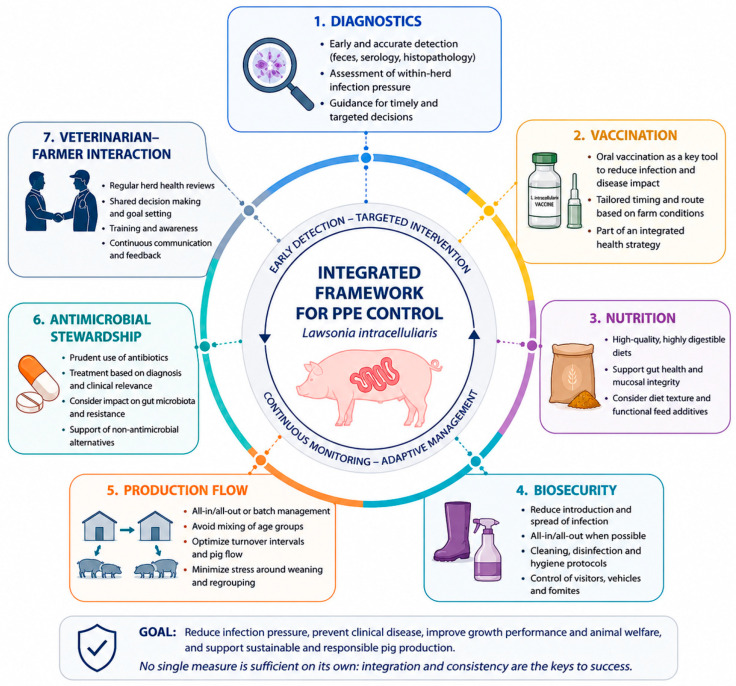
Integrated framework for the control of porcine proliferative enteropathy (PPE) caused by *Lawsonia intracellularis*. The framework highlights the seven key pillars that should work synergistically to reduce infection pressure, prevent clinical disease and minimize production losses: (1) diagnostics, enabling early and accurate detection of infection; (2) vaccination, with tailored timing according to herd epidemiology; (3) nutritional management, aimed at maintaining gut health and resilience; (4) biosecurity, to prevent pathogen introduction and within-herd spread; (5) production flow management, including all-in/all-out systems and optimized pig flow; (6) antimicrobial stewardship, promoting the responsible and targeted use of antimicrobials; and (7) veterinarian–farmer interaction.

Although the considerations presented in this article are supported by published literature and extensive field experience, the authors acknowledge that several aspects discussed are primarily based on clinical observations and expert opinion derived from endemic production systems. In addition, the availability of standardized epidemiological and productive data remains limited in many commercial settings, making direct comparisons among farms and production systems inherently difficult. Further longitudinal and harmonized field studies are therefore needed to better quantify the long-term impact of subclinical PPE and to optimize integrated control strategies under different production conditions. A schematic overview of the integrated approach proposed by the authors for PPE control is presented in Figure 1.

The figure was created by the authors using OpenAI DALL·E (GPT-5.5, OpenAI, San Francisco, CA, USA) and subsequently reviewed and edited for scientific accuracy and graphical layout.

## 4. Conclusions

The management of *L. intracellularis* infection represents a challenge of growing relevance in swine production, characterized by a level of complexity that clearly requires an integrated approach. In our opinion, the main gap in current PPE control is not the lack of effective preventive tools, but the persistent under-recognition of subclinical infection, which delays intervention and leads to an underestimation of its true health and economic impact. Addressing this gap requires a shift from reactive disease management toward proactive prevention.

Among the available control measures, vaccination against *L. intracellularis* represents a cornerstone of this integrated approach. We therefore propose that vaccination should no longer be considered an isolated preventive intervention, but rather a strategic tool whose effectiveness depends on its integration with diagnostics, herd management, and continuous epidemiological assessment. Timely implementation of diagnostic protocols and precise vaccine positioning based on the actual timing of infection within each herd are therefore essential, and vaccination strategies should always be customized according to the specific characteristics of individual farms.

The central message of this Opinion is that successful PPE control requires a change in perspective. Greater awareness of the hidden burden of subclinical infection, combined with stronger preventive strategies and continuous collaboration between veterinarians and farmers, is essential to improve disease recognition and promote the adoption of integrated control measures.

## Data Availability

The original contributions presented in this study are included in the article. Further inquiries can be directed to the corresponding author.
